# Underlying Factors Associated with Anemia in Amazonian Children: A Population-Based, Cross-Sectional Study

**DOI:** 10.1371/journal.pone.0036341

**Published:** 2012-05-04

**Authors:** Marly A. Cardoso, Kézia K.G. Scopel, Pascoal T. Muniz, Eduardo Villamor, Marcelo U. Ferreira

**Affiliations:** 1 Department of Nutrition, School of Public Health, University of São Paulo, São Paulo, Brazil; 2 Department of Parasitology, Immunology and Microbiology, Federal University of Juiz de Fora, Juiz de Fora, Minas Gerais, Brazil; 3 Department of Health Sciences, Federal University of Acre, Rio Branco, Acre, Brazil; 4 Departments of Environmental Health Sciences and Epidemiology, University of Michigan School of Public Health, Ann Arbor, Michigan, United States of America; 5 Department of Parasitology, Institute of Biomedical Sciences, University of São Paulo, São Paulo, Brazil; Barcelona Centre for International Health Research/Hospital Clinic/IDIBAPS/University of Barcelona, Spain

## Abstract

**Background:**

Although iron deficiency is considered to be the main cause of anemia in children worldwide, other contributors to childhood anemia remain little studied in developing countries. We estimated the relative contributions of different factors to anemia in a population-based, cross-sectional survey.

**Methodology:**

We obtained venous blood samples from 1111 children aged 6 months to 10 years living in the frontier town of Acrelândia, northwest Brazil, to estimate the prevalence of anemia and iron deficiency by measuring hemoglobin, erythrocyte indices, ferritin, soluble transferrin receptor, and C-reactive protein concentrations. Children were simultaneously screened for vitamin A, vitamin B_12_, and folate deficiencies; intestinal parasite infections; glucose-6-phosphate dehydrogenase deficiency; and sickle cell trait carriage. Multiple Poisson regression and adjusted prevalence ratios (aPR) were used to describe associations between anemia and the independent variables.

**Principal Findings:**

The prevalence of anemia, iron deficiency, and iron-deficiency anemia were 13.6%, 45.4%, and 10.3%, respectively. Children whose families were in the highest income quartile, compared with the lowest, had a lower risk of anemia (aPR, 0.60; 95%CI, 0.37–0.98). Child age (<24 months, 2.90; 2.01–4.20) and maternal parity (>2 pregnancies, 2.01; 1.40–2.87) were positively associated with anemia. Other associated correlates were iron deficiency (2.1; 1.4–3.0), vitamin B_12_ (1.4; 1.0–2.2), and folate (2.0; 1.3–3.1) deficiencies, and C-reactive protein concentrations (>5 mg/L, 1.5; 1.1–2.2).

**Conclusions:**

Addressing morbidities and multiple nutritional deficiencies in children and mothers and improving the purchasing power of poorer families are potentially important interventions to reduce the burden of anemia.

## Introduction

Iron deficiency (ID) is the most common and widespread nutritional deficiency worldwide. Because global prevalence estimates are not available for ID, anemia is often used as a proxy of ID and iron-deficiency anemia (IDA). Globally, half of the burden of childhood anemia is thought to be caused by ID [1]. Dietary inadequacies during the first two years of life, a critical period when children switch from a predominantly milk-based diet to a diet based on solid foods and require more iron for growth [2], are major determinants of anemia in young children. As a consequence, the WHO guidelines for primary prevention of anemia in young children have focused on iron supplementation [3]. However, the relative contribution of ID to childhood anemia varies widely across populations and age groups [4], [5]. Additional factors, such as infections, glucose-6-phosphate dehydrogenase (G6PD) deficiency, and hemoglobinopathies, are understudied contributors to anemia in tropical settings.

Recent socioeconomic development, with increased family income, wider access to education, and better income distribution, has led to a major decrease in the prevalence of child stunting in Brazil [6]. However, no clear downward trends have been observed for the prevalence of childhood anemia in this country [1]. Data from the most recent countrywide Demographic and Health Survey, carried out between 2006 and 2007, revealed that one-fifth of Brazilian children under the age of 5 years are anemic [7].

The prevalence of childhood anemia due to ID in Brazil remains largely unknown because hemoglobin (Hb) measurements used in nationwide representative surveys are neither sensitive nor specific as a screening test for ID. Lack of sensitivity occurs because a large proportion of total body iron must be lost before Hb concentrations fall below the conventional cut-off points to define anemia [8]. Low specificity is due to the presence of other causes of anemia, such as infection [9]–[11]. Many iron status indicators are affected by conditions commonly seen in children living in the tropics. Microcytosis, for example, may be caused by hemoglobinopathies [12]. Plasma ferritin (PF) concentration, the most specific biochemical correlate of total body iron stores, increases in infectious and inflammatory diseases, as do many other acute-phase proteins, including C-reactive protein (CRP) [8]. In contrast, concentrations of soluble transferrin receptor (sTfR), a sensitive indicator of ID at the tissue level, remain unaltered in inflammation and in most, although not all, infections [8].

In the present investigation, Hb, PF, sTfR, and CRP measurements were combined to determine the iron status of Amazonian children between 6 months and 10 years of age. In this population-based, cross-sectional study, children were simultaneously screened for vitamin A, vitamin B_12_, and folate deficiencies; intestinal parasite infections; G6PD deficiency; and sickle cell trait carriage. The aim of the study was to estimate the relative contribution of ID and other nutritional and non-nutritional factors to anemia in this population to provide a basis for public health intervention.

## Methods

### Ethical Statement

This study protocol was approved by the institutional review board of the School of Public Health, University of São Paulo, Brazil (No. 1681/07). Written informed consent was obtained from the parents or guardians of children who participated in the study. Data were analyzed anonymously.

### Study Design and Population

A population-based, cross-sectional study of children’s nutrition and health was carried out in December 2007 in Acrelândia, a frontier town situated 112 km east of Rio Branco, the capital of the state of Acre, western Brazilian Amazonia. The adult inhabitants of Acrelândia (urban population, 6000) are mainly migrants from southeast and south Brazil who are currently engaged in commercial agriculture and cattle raising. Infant mortality in Acrelândia, estimated at 70.7 per thousand live births in 2000, is substantially higher than the country average (27.7 per thousand live births) [13].

Sampling strategies, questionnaires, and field procedures were similar to those of a previous survey carried out in the same area in 2003 [14]. Briefly, a census of all households in the urban area of Acrelândia was performed, and 749 households with children younger than 10 years of age were identified. A total of 1225 children living in 734 households (98.0% of those identified in the census) were enrolled; mothers or guardians declined participation in 13 households, and two additional houses were closed. A questionnaire was administered through a face-to-face interview to the mothers or guardians of 1151 children (94.0% of those eligible). The interview, carried out during home visits by trained fieldworkers, addressed the following topics: (a) demographic characteristics (child’s sex, age, and race/ethnicity, classified as white, black, “pardo" [brown], yellow, or indigenous according to skin color as used in the Brazilian census) [15]; (b) socioeconomic status (ownership of household assets, paternal schooling, presence of the father in the household, maternal occupation, and number of inhabitants in the household) and environmental conditions (water supply, sewage disposal, and garbage disposal); (c) reproductive health variables (maternal age, child’s gestational age at birth, and birth weight); (d) nutritional history (duration of total and exclusive breastfeeding and age at introduction of weaning foods); and (e) morbidity (diarrhea, cough, or fever up to 15 days prior to the interview; episodes of malaria and wheezing in the past 12 months; and past hospitalization). The questionnaire was pilot-tested for clarity in a sample of the target population and revised accordingly before use in the study. After the interview, mothers and children were invited to visit the local family health clinic, where research clinicians carried out a physical examination and trained phlebotomists obtained a venous blood sample. During the interview, the fieldworker also provided participants with a plastic container, labeled with each child’s name and study code, and asked the mothers and guardians to collect a stool sample to be examined for intestinal parasites.

### Anthropometric Measurements

Length/height and weight were measured during the clinic visit by trained research assistants following standardized procedures and using calibrated equipment [16]. Among children <24 months of age, recumbent length was measured to the nearest millimeter with a locally made infant measuring board; weight was measured to the nearest 10 g with an electronic pediatric scale (model 1583; Tanita, Tokyo, Japan). Children 2 years or older were measured to the nearest 0.1 cm with a stadiometer (model 208; SECA, Hamburg, Germany) affixed to a flat surface on a wall, without a baseboard and perpendicular to the floor. The children were placed barefoot in a standing position in the middle of the stadiometer, with head, shoulders, buttocks, and heels pressed back against the wall; weight readings were taken on an electronic scale (model HS-302; Tanita, Tokyo, Japan) and recorded to the nearest 100 g. Each measurement was repeated and the mean value calculated. Birth date was recorded directly from birth certificates or child health cards. Z-scores for length/height-for-age (HAZ) were calculated according to the WHO child growth standards [17] for children aged 0 to 5 years and the WHO Growth Reference Data [18] for children >5 years. Stunting was defined as a HAZ of <–2.0 [16].

### Laboratory Analysis

A sample (approximately 5 mL) of fasting venous blood was collected from 1111 children aged between 6 months and 10 years (90.7% of those enrolled). Because limited sample volumes were available, not all laboratory analyses were performed for all children. At the field laboratory in Acrelândia, whole blood aliquots collected in EDTA-containing vacuum tubes were used to carry out full blood cell counts and measure Hb concentrations on an automated cell counter (ABX Micro 60; Horiba, Montpellier, France). A separate blood sample was protected from light and centrifuged within 1 h of collection; serum and plasma samples were shipped to São Paulo on dry ice and frozen at –70°C until further analysis. In São Paulo, PF and sTfR concentrations were measured using commercially available enzyme immunoassays (Ramco, Houston, TX); CRP concentrations were measured using a high-sensitivity chemiluminescent assay (Immulite; DPC, Los Angeles, CA). Anemia, ID, and IDA were defined according to Hb, PF, sTfR, and CRP concentrations as follows [3]: Anemia was defined as an Hb concentration of <110.0 g/L (children 6–59 months of age) or <115.0 g/L (children >60 months of age). ID was defined when PF concentrations were low (<12 µg/L for children <5 years of age or <15 µg/L for those >5 years of age) or when sTfR concentrations were high (>8.3 mg/L). IDA was defined when ID occurred in anemic children. Because inflammation or infection may increase PF concentrations in iron-deficient children, concentrations of the acute-phase protein CRP were employed to estimate the CRP-containing index, defined as (0.34+0.0043×PF – [2.7×sTfR]/PF+0.00696×CRP+0.05×sTfR). Children with a CRP-containing index below zero were also considered to have ID [19], [20]. The cut-off CRP level of >5 mg/L was considered to be associated with inflammation as described in a recent meta-analysis [21]. Serum folate and vitamin B_12_ concentrations were measured using commercial fluoroimmunoassays (Perkin Elmer, Wallac Oy, Turku, Finland); values <10 nmol/L and <150 pmol/L were considered diagnostic of folate and vitamin B_12_ deficiency, respectively [22]. Serum concentrations of vitamin A (retinol) were measured by standard HPLC methods [23]; concentrations of <0.70 µmol/L were considered diagnostic of vitamin A deficiency [24]. Frozen samples were analyzed within 6 months of collection. Children with anemia or nutrient deficiencies detected during the survey received adequate treatment prescribed by the medical team involved in the project in accordance with WHO guidelines [2], [22], [24].

DNA-based tests were used to screen for G6PD deficiency and sickle cell trait carriage. DNA was extracted from EDTA-containing whole blood samples using DNA kits (Qiagen, Hilden, Germany). The G6PD202A mutation (rs1050828) was genotyped, constituting the most frequent diagnostic single-nucleotide polymorphism associated with G6PD deficiency found in the Amazon Basin of Brazil [25]. Boys who were hemizygous and girls that were homozygous for the G6PD202A allele were considered to be G6PD-deficient. The sickle mutation (HbS, rs334) in the β-globin gene was also genotyped to detect carriers of the sickle cell trait (heterozygotes for HbS) and sickle cell disease (homozygotes for HbS). Genotyping was carried out by allele-specific PCR with molecular beacons, under contract, by Prevention Genetics (Marshfield, MA). The internal quality of genotypic data was assessed by typing 10% of blinded samples in duplicate; agreement was >99%.

Stool samples were collected in plastic containers containing a preservative solution (10% formalin) from 989 children aged between 6 months and 10 years (80.7% of those enrolled). Logistic limitations prevented the collection of more than one stool sample from each participant, but prevalence estimates derived from examination of a single sample have been shown to be accurate for most infections with soil-transmitted helminths (geohelminths) associated with anemia [26]. Because variable volumes of feces were mixed with formalin for preservation, no attempt was made to perform egg counts. The stool samples were examined for parasite ova, cysts, and larvae as described elsewhere [14]. Geohelminths found in this population included *Ascaris lumbricoides, Trichuris trichiura,* and *Strongyloides stercoralis.* Children with intestinal parasitic infections received free treatment prescribed by research clinicians.

### Statistical Analysis

Principal component analysis was used to derive a wealth index, a proxy of household income [27], from information on ownership of 12 household assets as described elsewhere [14]. In most of the analyses, children were grouped into three age categories: infants and toddlers (6–23 months of age), preschool children (24–59 months of age), or school children (5–10 years of age). Multiple Poisson regression was used with robust variance to estimate adjusted prevalence ratios (PR) for independent associations with anemia according to demographic, socioeconomic, environmental, and morbidity covariates. To this end, a hierarchical model with three concentrations of determination was used [28]: (i) socioeconomic and demographic variables (wealth index, race/ethnicity, maternal schooling, maternal age, and parity); (ii) birth and perinatal variables (birth weight and gestational age) and breastfeeding practices; and (iii) reported morbidity and conditions diagnosed by laboratory analysis. At each level of determination, covariates were retained in the model if they were associated with the outcome at *P*<0.10 and for ordinal variables when they followed a dose-response pattern or if their inclusion in the model changed the PR by 10% or more. Missing observations were included in the multiple models by creating missing-value categories. Potential correlations among siblings within the same household were assessed in multilevel regression models using the STATA “xtmepoisson" procedure. Estimates of the population attributable fraction for dichotomous exposures were calculated using the following formula: attributable fraction = p (PR-1)/PR, where p is the proportion of cases exposed, and PR is the adjusted PR [29]. In supplemental analyses, we carried out multiple linear regression to describe independent associations between concentrations of Hb, PF, and sTfR (dependent variables) and demographic, socioeconomic, and morbidity covariates; natural log transformations of PF, sTfR, and CRP were used to fit the models. All p-values reported are two-tailed. Statistical analyses were performed using STATA version 11.0 (College Station, TX).

## Results

Of the 1151 participants, 1111 children (97%) >6 months of age provided blood samples and constituted the analytic sample. On average, there were 1.7 participants per household, ranging from 1 to 5 children. No sanitation system was available in the town. Among those identified as either head of the household or the primary caregiver, 38.1% had studied for <5 years. The race/ethnicity of the majority of the participants was classified as brown (86%); a high proportion of mothers with only primary school education (38%) and a history of more than two pregnancies (64%) was observed (Table 1).

**Table 1:** General characteristics of children aged 6 months to 10 years (N = 1111).

Variable	Value^a^
	*n* (%)
**Sex**	
** Female**	559 (50.3)
** Male**	552 (49.7)
**Race/ethnicity**	
White	98 (9.5)
Black	52 (5.0)
Brown	882 (85.5)
**Maternal schooling**	
≤4 years	423 (38.1)
5–8 years	319 (28.7)
≥9 years	331 (29.8)
**Maternal pregnancies**	
≤2	378 (34.0)
≥3	712 (64.1)
**Maternal age**	
10–21 years	106 (9.5)
22–35 years	743 (66.9)
>35 years	262 (23.6)
**Age at introduction of cow milk**	
<90 d	300 (27.0)
≥90 d	655 (64.1)
**Diarrhea in the last 15 d**	
No	840 (75.6)
Yes, 1–3 d	178 (16.0)
Yes, ≥4 d	89 (8.0)

a Totals differ from the total number of study children because of missing values.

Median values and interquartile ranges for iron status indicators and prevalence of anemia and other conditions are shown in Table 2. The overall prevalence of anemia was 13.6%; the highest rates were found in infants and toddlers (40%), and no significant differences were found between sexes. No cases of severe anemia (Hb <70 g/L) were diagnosed, and only 1.5% of the study subjects had Hb ≤100 g/L. Evidence for ID (altered PF, sTfR, or both) was found in 45% of subjects, with the highest prevalence among children under the age of 2 years. A total of 76% of all anemic children had evidence of ID, whereas the proportion was 94% among infants and toddlers. The overall prevalence of IDA was 10.3%, and only 23% of subjects with ID were anemic. The overall prevalence rates of stunting and deficiencies of retinol, vitamin B_12_, and folate were 5.5%, 14.2%, 3.7%, and 2.5%, respectively. Only one case of sickle cell disease was observed. Of the 1034 children for whom plasma CRP information was available, only 41 (4%) had CRP values of ≥10 mg/L. The CRP index yielded similar percentage estimates of ID in all age groups.

**Table 2:** Iron status indicators and prevalence of anemia and other nutritional and non-nutritional conditions in urban Amazonian children^a^.

	Infants and toddlers,6–24 mo (n = 190)	Pre-school children,25–59 mo (n = 336)	School children,5–10 y (n = 585)	Overall (n = 1111)	
**Hb (g/L)**					
Median (IQR)	112.0 (104.0; 120.0)	122.0 (115.0; 128.0)	127.0 (122.0; 133.0)	123.0 (115.0; 131.0)	
% (95% CI) below cut-off^b^	40.0 (33.0 – 47.3)	10.4 (7.4–14.2)	6.8 (4.9–9.2)	13.6 (11.6–15.7)	
**Mean corpuscular volume (fl)**					
Median (IQR)	72 (67; 76)	79 (75; 81)	82 (80; 84)	80 (76; 83)	
% (95% CI) below cut-off^c^	22.5 (16.7 – 29.1)	15.0(11.3 – 19.3)	3.8(2.4 – 5.6)	10.3(8.6 – 12.3)	
**Plasma ferritin (PF, µg/L)**	[183]	[294]	[582]	[1094]	
Median	12.2 (5.9; 20.2)	26.9 (13.7; 46.8)	45.3 (30.9; 65.7)	34.5 (17.8; 54.8)	
% (95% CI) below cut-off^d^	48.9 (41.5–56.4)	22.5 (18.1–27.4)	5.0 (3.4 – 7.1)	17.6 (15.4 – 20.0)	
**Soluble transferrin receptor** **(sTfR, mg/L)**	[183]	[294]	[582]	[1094]	
Median (IQR)	10.8 (8.6; 14.0)	8.1 (6.8; 9.6)	7.1 (6.1; 8.2)	7.7 (6.5; 9.4)	
% (95% CI) above cut-off^e^	79.9 (73.3 – 85.4)	47.1 (41.6 – 52.7)	23.1 (19.7 – 26.7)	39.9 (36.9 – 42.8)	
**CRP (mg/L)**	[170]	[305]	[559]	[1034]	
Median (IQR)	0.70 (0.21; 3.00)	0.39 (0.01; 1.21)	0.39 (0.01; 1.05)	0.43 (0.01; 1.31)	
% (95% CI) above cut-off^f^	28.1 (21.5 – 35.4)	17.0 (13.0 – 21.7)	13.3 (10.6 – 16.4)	16.8 (14.6 – 19.2)	
CRP-index <0^g^	86.0 (79.8–90.8) [170]	44.9 (39.2 – 50.7) [294]	14.9 (12.0–18.1) [559]	35.5 (32.6–38.5) [1023]	
**Vitamin A (µmol/L)**	[165]	[302]	[561]	[1028]	
Median (IQR)	1.13 (0.88; 1.50)	1.17 (0.88; 1.52)	1.14 (0.87; 1.46)	1.15 (0.87 ; 1.50)	
% (95% CI) below cut-off^h^	13.3 (8.5–19.5)	13.9 (10.2–18.3)	14.6 (11.8–17.8)	14.2 (12.1–16.5)	
**Vitamin B-12 (pmol/L)**	[159]	[291]	[517]	[967]	
Median (IQR)	244 (181; 308)	279 (225; 364)	250 (208; 306)	258 (209; 320)	
% (95% CI) below cut-off^i^	12.0 (7.4–18.0)	2.8 (1.2–5.3)	1.7 (0.8–3.3)	3.7 (2.6–5.1)	
**Folate (nmol/L)**	[167]	[305]	[560]	[1032]	
Median (IQR)	22.1 (17.0; 32.2)	23.0 (17.4; 30.0)	23.6 (18.1; 29.8)	23.3 (17.7; 30.3)	
% (95% CI) below cut-off^j^	4.2 (1.7–8.4)	2.3 (0.9–4.7)	2.1 (1.1–3.7)	2.5 (1.7–3.7)	
**Prevalence of selected conditions**, % (95% CI)					
Iron deficiency^k^	84.8 (78.8–89.6) [183]	55.0 (49.5–60.5) [294]	26.7 (23.1 – 30.5) [582]	45.0 (42.0–48.0) [1094]	
Iron deficiency anemia	37.5 (30.5–45.0) [183]	8.5 (5.7–12.1) [294]	2.8 (1.6 – 4.4) [582]	10.3 (8.6–12.2) [1094]	
Stunting	11.8 (7.5–17.3)	4.5 (2.5–7.3)	3.8 (2.4–5.7)	5.4 (4.1–6.9)	
Geohelminth infection^l^	2.4 (0.6–5.9) [169]	2.4 (0.9–4.8) [297]	5.2 (3.4–7.4) [523]	3.8 (2.7–5.2) [989]	
G6PD deficiency	7.0 (3.7–11.9) [170]	10.4 (7.2–14.3) [308]	8.6 (6.4–11.3) [536]	8.9 (7.2–10.8) [1104]	
Sickle cell trait carriage	3.9 (1.6–7.9) [178]	5.3 (3.1–8.3) [321]	3.9 (2.5–5.8) [565]	4.3 (3.2–5.7) [1064]	

IQR, interquartile ranges.

a Totals in brackets differ from the total number of study children by age group because of missing values.

b Cut-offs for anemia: <110.0 and <115.0 g/L for 6–59 months and ≥60 months, respectively;

c Cut-offs for microcytosis by age: <67, <73, <74, and <76 fl for <24 months, 24–59 months, 5–7.9 years, and 8–11.9 years, respectively;

d PF: <12 and <15 µg/L for <59 and ≥60 months, respectively;

e sTfR: >8.3 mg/L;

f Cut-off for high CRP: >5 mg/L;

g CRP index defined as (0.34+0.0043×PF – [2.7×sTfR]/PF+0.00696×CRP+0.05×sTfR);

h Serum retinol <0.70µmol/L;

i Serum vitamin B_12_<150 pmol/L;

j Serum folate <10 nmol/L;

k According to cut-offs for PF or sTfR.

l Geohelminths in this population included *Ascaris lumbricoides* (overall prevalence, 2.4%), *Strongyloides stercoralis* (0.5%), and *Trichuris trichiura* (0.8%) - the same subject may be co-infected with more than one species.

Infants and toddlers had the highest prevalence of anemic children with coexistence of two or more nutrient deficiencies (17.5%) compared with preschool and school children (2.4 and 1.4, respectively; *P*<0.05, χ^2^ test). Microcytosis (low mean corpuscular volume as defined in Table 2) was observed in 56%, 31% and 15% of children with IDA, folate and vitamin B12 deficiency, respectively. Anemia was uniformly prevalent according to race/ethnicity, birth conditions, and breastfeeding practices. The most common reported morbidities over the preceding 15 days were diarrhea (overall, 23.4%; <2 years, 46.0%) and wheezing (overall, 10.7%; <2 years, 22.3%). Twelve cases of malaria and one case of dengue fever over the previous year were reported. The use of antihelminthics over the past 6 months was reported by the caregivers for 35.4% of the participants, with a higher frequency of use among preschool children (43.5%) (data not shown in Tables).

The relative contribution of each risk factor to anemia was estimated from adjusted prevalence ratios (aPR) derived from multiple Poisson regression models with anemia as the outcome. Children living in families that belonged to a higher wealth index quartile were at lower risk for anemia (adjusted PR, 0.60; 95%CI, 0.37–0.98). In contrast, children <2 years of age (2.90; 2.01–4.20) and children whose mothers had more than two pregnancies (2.01; 1.40–2.87) were at higher risk for anemia. Other associated risk factors were ID, deficiency of folate or vitamin B_12_, and high CRP concentrations (>5 mg/L). Attributable fractions were estimated for factors other than age, sex, wealth index, maternal schooling, and maternal age (Table 3). Overall, 5.3% and 4.8% of all cases of anemia were attributable to ID and to high parity of mothers (more than two pregnancies), respectively. The corresponding figures among children <2 years of age were 16.8% and 7.9%, respectively (Table 3). Similar results from multiple models were found with additional adjustment for household on multilevel analysis (data not shown).

In addition to age and wealth index, circulating concentrations of sTfR, retinol, vitamin B_12_, and CRP were strong independent predictors of Hb concentrations. The final model explained 37.6% of the variance of Hb concentration in the children studied. On average, (a) increases of 1 mg/L of sTfR and 2.72 mg/L of CRP (1 log unit) were associated with 1.21 and 0.31 g/L decreases in Hb, respectively; (b) decreases of 1 µmol/L of retinol and 1 pmol/L of vitamin B_12_ were associated with 1.71 and 0.01 g/L decreases in Hb, respectively (Table 4). In separate analysis, the predictors of sTfR were examined in linear models (results not shown in Tables). In addition to age and sex, log CRP concentrations were positively associated with plasma sTfR (β = 0.021; 95%CI, 0.013–0.028; *P* = 0.000).

**Table 3:** Attributable fractions (AF) of anemia according to age group in urban Amazonian children.

	Infants and toddlers,6–24mo (n = 190)		Pre-school children,25–59 mo (n = 336)		School children,5–10 y (n = 585)		Overall(n = 1111)	
**Risk factors**	**p** a **aPR(95%CI)** b	**AF** c **%**	**p** a **aPR(95%CI)** b	**AF** c **%**	**p** a **aPR(95%CI)** b	**AF** c **%**	**p** a **aPR(95%CI)** b	**AF** c **%**
High parity (≥3 pregnancies)	22.2 1.55 (1.03–2.33)	7.9	7.7 3.24 (1.48–7.08)	5.3	–	–	9.5 2.01 (1.40–2.87)	4.8
Iron deficiency	37.2 1.82 (0.93–3.59)	16.8	8.5 3.41 (1.54–7.51)	6.0	2.9 1.96 (1.08–3.53)	1.4	10.3 2.06 (1.40–3.04)	5.3
Folate deficiency	4.2 2.46 (1.62–3.72)	2.5	0.7 3.63 (1.34–9.82)	0.5	–	–	0.9 2.00 (1.27–3.13)	0.5
Vitamin B-12 deficiency	7.6 1.75 (1.13–2.71)	3.3	–	–	–	–	1.4 1.44 (0.95–2.19)	0.4
High plasma CRP (>5 mg/L)	10.0 1.42 (0.90–2.23)	3.0	–	–	–	–	3.1 1.53 (1.08–2.17)	1.1

a Prevalence (%) of cases exposed in each risk factors.

b Adjusted prevalence ratio (aPR) estimated from multiple Poisson regression models with additional adjustment for age (in overall analysis), sex, wealth index (quartile), maternal schooling (≤4, 5–8, and ≥9 years), and maternal age (10–21, 22–35, and >35 years).

c Attributable fraction defined as p(aPR –1)/aPR.

**Table 4:** Multiple linear regression analysis of factors associated with hemoglobin in urban Amazonian children.

Dependent variable	Independent variable	β	95% CI	*P*	R^2^	n^a^
Hemoglobin (g/L)					0.376	943
	Age (months)	0.114	0.095; 0.133	0.000		
	Wealth index (continuous)	0.238	0.079; 0.397	0.003		
	sTfR (mg/L)	–1.209	–1.419; –0.998	0.000		
	Serum Vitamin B-12 (pmol/L)	0.013	0.007; 0.019	0.000		
	Serum retinol (µmol/L)	1.713	0.552; 2.875	0.004		
	Log CRP (mg/L)	–0.310	–0.574; –0.045	0.022		

a Total differs from total number of study children because of missing values.

## Discussion

Anemia in the Amazonian children studied was twice as prevalent as that in comparable population groups from industrialized countries [1], [30], but remained less frequent than that in most of the developing world [4], [31]. In line with rates reported by previous studies (31,32), more than 50% of anemic young children had evidence of ID, but only 5% of all cases of anemia in the overall population were attributable to ID. Among children under 2 years of age, 17% of all cases of anemia were attributable to ID along with deficiencies in vitamin B_12_ and folate and high plasma CRP (approximately 3% for each risk factor).

Although the overall prevalence of vitamin B_12_ and folate deficiency found in this study (3.7% and 2.5%, respectively) was low, vitamin B_12_ deficiency was observed in 12% of examined children under the age of 2 years and was associated with anemia. Comparisons with available data within and between countries are difficult because the majority of data on the prevalence of folate and vitamin B_12_ deficiencies are derived from relatively small surveys with different nutritional status indicators. Anemia due to folate and vitamin B_12_ deficiency has been considered uncommon worldwide. This is probably due to limitations in diagnosis because iron deficiency tends to lower the mean corpuscular volume (MCV) in the complete blood count more readily than it is raised by folate or vitamin B12 deficiency while the use of other biochemical indicators is expensive [22]. A study of 2800 children aged 5 to 12 years from Bogota’s public schools in Colombia found vitamin B_12_ deficiency (<148 pmol/L) in 1.6% of children. Plasma vitamin B_12_ concentrations were inversely related to the mother’s parity and positively associated with socioeconomic status and a dietary pattern that included frequent intake of beef, chicken, and dairy products in a dose-response manner [33]. An earlier study of 12- to 23-month-old toddlers who lived in rural India found that 2.3% of children had vitamin B_12_ deficiency alone (<150 pmol/L) and 66% had at least one micronutrient deficiency (vitamin B_12_, folate, iron, or vitamin A) [34]. One explanation for the higher prevalence of vitamin B_12_ deficiency among toddlers in the present study might be related to low daily intakes of vitamin B_12_ as described in a previous study in the Amazon area [35]. The diet of young Amazonian children tends to comprise few foods in small amounts. The dietary pattern reflects a high intake of carbohydrate-rich foods and cow milk, with irregular intakes of fruit, vegetables, and meat. Compulsory fortification of flours with iron and folate, in place in Brazil since 2003, has been shown to be less effective in children under 2 years of age because their flour consumption may not be sufficient to meet the required intake of key micronutrients [36].

The high prevalence (14.2%) of vitamin A deficiency observed in our population of a similar magnitude across all age groups is of particular concern. Although this vitamin A deficiency was not found to be associated with anemia, a positive association between serum retinol and Hb concentration was observed, as reported by other authors in studies of children in Latin America and elsewhere [4], [37]. Although the role of vitamin A in iron status has been described in the literature [38], [39], the effect of suboptimal vitamin A status on the prevalence of anemia has not been considered in public health programs to prevent and control anemia in children. Several lines of evidence indicate that vitamin A metabolism is altered in situations of iron deficiency, with low serum retinol concentrations and increased hepatic retinyl ester or retinol stores. These changes probably result from a reduction in the activity of retinyl ester hydrolases with a consequent decrease in vitamin A mobilization, or from an increase in retinol sequestration to the liver [39]. It seems that restricted access to diverse micronutrient-rich diets can exacerbate nutritional anemias. Thus, multiple deficiencies and frequent exposure to endemic disease tend to cluster within individuals, and the synergistic effect of these deficiencies is important in the development of anemia [40].

As expected, the high frequency of morbidities observed in this study population increased the concentrations of both PF and sTfR [11], [41]; the effect on sTfR may be due to true infection-associated ID or increased erythropoiesis following hemolysis [8]. These effects on PF and sTfR complicated the analysis of the contribution of morbidities to ID in our population, despite its relatively high attributable fraction. Geohelminth infection was infrequent (overall prevalence, 3.8%), and the impact of geohelminth control on the iron status of our population, although not negligible, is likely to be less pronounced than that in African [11] or Asian [4], [9] populations. Previous surveys of risk factors for anemia have implicated ID and malaria as strong predictors of anemia (prevalence, 9%–30%) in rural Amazonian communities, with no significant contribution of geohelminth infection [42], [43]. The estimated prevalence of G6PD deficiency is <10% in this and other Amazonian populations [44]. G6PD-deficient subjects are prone to hemolysis induced by primaquine, an antimalarial drug used in this area without screening, further aggravating malaria-related anemia [45]. However, because malaria infection was uncommon in the present study, G6PD deficiency was not associated with anemia risk. Sickle-cell disease is unlikely to represent a major cause of anemia in our area; low HbS allele frequencies (<5%) were found in this and other Amazonian populations with a similar ethnic composition [46].

Previous studies have demonstrated that the role of anemia of inflammation in the pathogenesis of anemia within the developing world plays a central role in the context of infectious diseases prevalent in these regions [47], [48]. Both IDA and anemia of chronic or acute disease is difficult to distinguish because increased circulating hepcidin seen during inflammation blocks iron release from enterocytes and the reticuloendothelial system, resulting in iron-deficient erythropoiesis [40]. In the present study population, children under 2 years of age showed higher median values of CRP with frequent exposure to comorbidities (mainly diarrhea and respiratory tract infections), conditions that may distort estimates of iron status. In age-adjusted models, CRP was inversely associated with Hb concentrations, suggesting inflammation as a predictor of Hb levels independent of the child’s age.

A body of evidence reinforces the concept that anemia is a marker of socioeconomic disadvantage, with the poorest and least educated being at greatest risk of exposure to risk factors for anemia and its sequelae [40]. In the present study, anemia was predicted by socioeconomic factors: the risk of anemia among children living in a household belonging to the lowest wealth quartile was 40% higher than among those in the highest wealth quartile. Higher wealth index values predicted higher Hb concentrations. In Brazil, child health has improved in recent decades, but this improvement has been less marked in the northern region, which encompasses the Amazon. Brazilian national household surveys carried out over a 33-year period have shown a steep decline in the overall prevalence of stunting among children under 5 years of age because of economic growth coupled with equity-oriented public policies and improvements in the population’s purchasing power, maternal education, sanitation, and access to health care [6]. However, declines of a similar magnitude have not been confirmed for the prevalence of anemia in Brazilian children. Trend data from selected Latin American countries with nationally representative data show that reductions in anemia are generally occurring slowly [32].

In our study, high maternal parity (more than 2 pregnancies) was positively associated with anemia. In developing countries this factor can be considered as a marker of early onset of childbearing, short intervals between births, and poor access to antenatal care and supplementation [49]. The intergenerational transfer of poor iron status from mother to child has been proposed as an additional factor to explain the vulnerability of infants to ID and anemia [40]. Thus, in addition to socioeconomic progress, strategies to lower the risk of anemia in early infancy should include optimization of maternal nutritional status and antenatal care, delayed cord clamping at delivery, improvement of complementary feeding practices, and prevention and treatment of infectious disease [40], [50].

Certain limitations in the present study should be noted. The principal constraint of our study is its cross-sectional design. Therefore, the temporal relation between exposures and outcomes (anemia or Hb concentrations) could not be established to ensure that micronutrient deficiencies and elevations in CRP concentrations precede the outcomes. Despite this limitation, we have identified a comprehensive set of associated factors with anemia and Hb concentrations in Amazonian children aged 6 months to 10 years. No other population-based surveys of anemia and ID in Brazil have used a wide-ranging combination of biochemical indicators; available national estimates of ID prevalence have mostly been based on Hb determination.

In conclusion, approximately 5% of all cases of anemia in our study population were attributable to ID; a similar attributable fraction was estimated for high parity (more than two pregnancies) among mothers. Among infants and toddlers, almost one in five cases of anemia was attributable to ID; purchasing power, serum concentrations of vitamin B_12_ and retinol, and elevated CRP concentrations as a measure of recent infection were additional factors shown to affect Hb concentrations. Strategies to control anemia based exclusively on iron supplementation are likely to have only a limited impact on the overall prevalence of anemia. Controlling morbidities and multiple nutritional deficiencies and improving maternal nutritional status and the purchasing power of poorer families represent potentially important interventions.
